# Amifostine alleviates radiation-induced lethal small bowel damage via promotion of 14-3-3σ-mediated nuclear p53 accumulation

**DOI:** 10.18632/oncotarget.2386

**Published:** 2014-10-31

**Authors:** Eng-Yen Huang, Feng-Sheng Wang, Yu-Min Chen, Yi-Fan Chen, Chung-Chi Wang, I-Hui Lin, Yu-Jie Huang, Kuender D. Yang

**Affiliations:** ^1^ Department of Radiation Oncology, Kaohsiung Chang Gung Memorial Hospital, Chang Gung University College of Medicine, 123 Ta-Pei Road, Niao-Sung District, Kaohsiung 833, Taiwan; ^2^ Graduate Institute of Clinical Medical Sciences, Chang Gung University College of Medicine, Taiwan; ^3^ Department of Medical Research, Kaohsiung Chang Gung Memorial Hospital, Chang Gung University College of Medicine, 123 Ta-Pei Road, Niao-Sung District, Kaohsiung 833, Taiwan; ^4^ Center for Laboratory Animals, Kaohsiung Chang Gung Memorial Hospital, Chang Gung University College of Medicine, 123 Ta-Pei Road, Niao-Sung District, Kaohsiung 833, Taiwan; ^5^ School of Traditional Chinese Medicine, Chang Gung University College of Medicine, Taiwan; ^6^ Department of Medical Research, Show Chwan Memorial Hospital in Chang Bing, 6-1 Lu-Kung Road, Chang Bing Industrial Center, Lu-Kang, Changhua 505, Taiwan; ^7^ Institute of Clinical Medicine, National Yang Ming University, Taiwan

**Keywords:** amifostine, p53, small bowel, whole-abdominal irradiation, 14-3-3σ, MDM2

## Abstract

Amifostine (AM) is a radioprotector that scavenges free radicals and is used in patients undergoing radiotherapy. p53 has long been implicated in cell cycle arrest for cellular repair after radiation exposure. We therefore investigated the protective p53-dependent mechanism of AM on small bowel damage after lethal whole-abdominal irradiation (WAI). AM increased both the survival rate of rats and crypt survival following lethal 18 Gy WAI. The p53 inhibitor PFT-α compromised AM-mediated effects when administered prior to AM administration. AM significantly increased clonogenic survival in IEC-6 cells expressing wild type p53 but not in p53 knockdown cells. AM significantly increased p53 nuclear accumulation and p53 tetramer expression before irradiation through the inhibition of p53 degradation. AM inhibited p53 interactions with MDM2 but enhanced p53 interactions with 14-3-3σ. Knockdown of 14-3-3σ also compromised the effect of AM on clonogenic survival and p53 nuclear accumulation in IEC-6 cells. For the first time, our data reveal that AM alleviates lethal small bowel damage through the induction of 14-3-3σ and subsequent accumulation of p53. Enhancement of the p53/14-3-3σ interaction results in p53 tetramerization in the nucleus that rescues lethal small bowel damage.

## INTRODUCTION

Radiotherapy plays an important role in definitive and adjuvant therapy for many cancers. However, radiotherapy-associated toxicity limits the delivery of large doses of radiation. In patients with pelvic malignancies, such as those in the uterus, rectum, bladder, or prostate, small bowel toxicities are common during radiotherapy. We noted that small bowel volume was correlated with acute diarrhea in patients with gynecological malignancies [1]. The effort to reduce small bowel toxicities is an important issue for pelvic radiotherapy.

Pharmacological intervention has been considered for the alleviation of small bowel toxicities, in addition to dosimetric modulation of the small bowel volume. Amifostine (AM) (WR-2721) is the only radioprotective drug approved by the FDA for head and neck cancer. Yuhas and Storer found that WR-2721 exerted a radioprotective effect in both the skin and bone marrow of mice without increasing the radioresistance of the solid tumors [2]. Milas et al. also noted that WR-2721 could protect the mouse jejunum [3]. However, it has been determined that WR-2721 should be administered before irradiation to exert its protective effect. The well-known radioprotective mechanism of WR-2721 occurs via scavenging of free radicals induced by radiation [4]. Murray et al. found that free radical scavenging by WR-2721 may contribute to but not fully explain the drug's protective action [5].

Understanding the key mechanism of radiation-induced acute small bowel damage would aid in determining the mechanism of AM-mediated protection. p53 is an important molecule for the control of acute small bowel damage after irradiation [6,7,8]. Interestingly, WR-1065, the active form of AM, binds to p53 [9]. Tetramerization of p53 leads to its transcriptional activation [10], and 14-3-3σ can increase p53 stabilization [11,12]. Therefore, we were interested in the role of p53 in radioprotection by AM. Here, we show that p53 and 14-3-3σ cooperate with AM-mediated resistance to radiation-induced small bowel damage.

## RESULTS

### Both AM and NAC attenuate initial oxidative DNA damage

**Figure 1:**
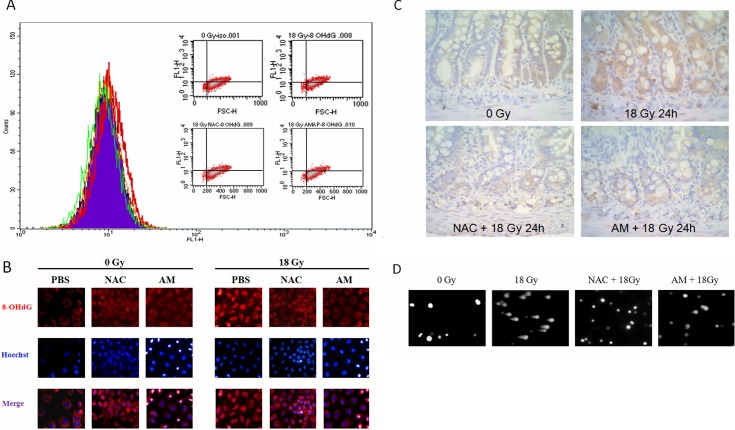
Equal effects of NAC and AM on oxidative DNA damage IEC-6 cells were incubated with NAC (4 mM) or AM (4 mM) for 60 minutes and irradiated (18 Gy). **(A)** Measurement of 8-OHdG among the 0 Gy (purple), 18 Gy (red), NAC plus 18 Gy (orange), and AM plus 18 Gy (green) groups was performed using flow cytometry. Cells were fixed 30 minutes after irradiation. **(B)** Localization of 8-OHdG was assessed by immunofluorescence between different groups before and after irradiation. Cells were fixed 30 minutes after irradiation. **(C)** Immunohistochemistry of 8-OHdG 24 hours after irradiation in rats pretreatment with vehicle, NAC (200 mg/kg), or AM (200 mg/kg) 30 minutes before 18 Gy whole-abdominal irradiation. Original magnification, 400×. **(D)** DNA damage was measured using a Comet assay. Cells were analyzed 5 minutes after irradiation.

To examine the antioxidant role of AM, we used N-acetylcysteine (NAC), an antioxidant thiocompound similar to AM, to compare radiation-induced damage between cells treated with these compounds. The compound 8-hydroxy-2-deoxyguanosine (8-OHdG) is a marker of oxidative DNA damage. We compared 8-OHdG levels using flow cytometry between vehicle-, AM-, and NAC-treated IEC-6 cells before and after 18 Gy irradiation, a lethal dose for the small bowel of rats. Increased 8-OHdG levels were noted 30 minutes after 18 Gy irradiation compared with levels following 0 Gy irradiation. NAC- or AM-pretreated IEC-6 cells showed decreased levels of 8-OHdG after 18 Gy irradiation that were similar to the levels in cells treated with 0 Gy (Figure 1A). Next, we used immunofluorescence staining to examine the localization of 8-OHdG in cells. The baseline distribution of 8-OHdG in unirradiated cells was in the cytosol in the PBS, NAC, and AM groups (Figure 1B). Irradiation of 18 Gy resulted in the accumulation of 8-OHdG in the nucleus at 30 minutes, and treatment with either NAC or AM attenuated the accumulation of 8-OHdG to levels observed in the 0 Gy-irradiated cells (Figure 1B). *In vivo* confirmation of oxidative stress in intestinal crypts was performed using immunohistochemistry (IHC) for 8-OHdG staining 24 hours after 18 Gy WAI in rats. Irradiation increased 8-OHdG staining in the crypts. NAC (200 mg/kg) or AM (200 mg/kg) administered 30 minutes before 18 Gy whole-abdominal irradiation (WAI) also equally alleviated oxidative stress in the crypts of the jejunum (Figure 1C). We next used a Comet assay to detect DNA damage in these three groups at 5 minutes after 18 Gy irradiation. The data revealed that 18 Gy irradiation-induced DNA damage resulted in long comet tails, while shortened tails were noted in IEC-6 cells pretreated with NAC or AM (Figure 1D).

### p53-dependent radioprotection of small bowel damage by AM

**Figure 2:**
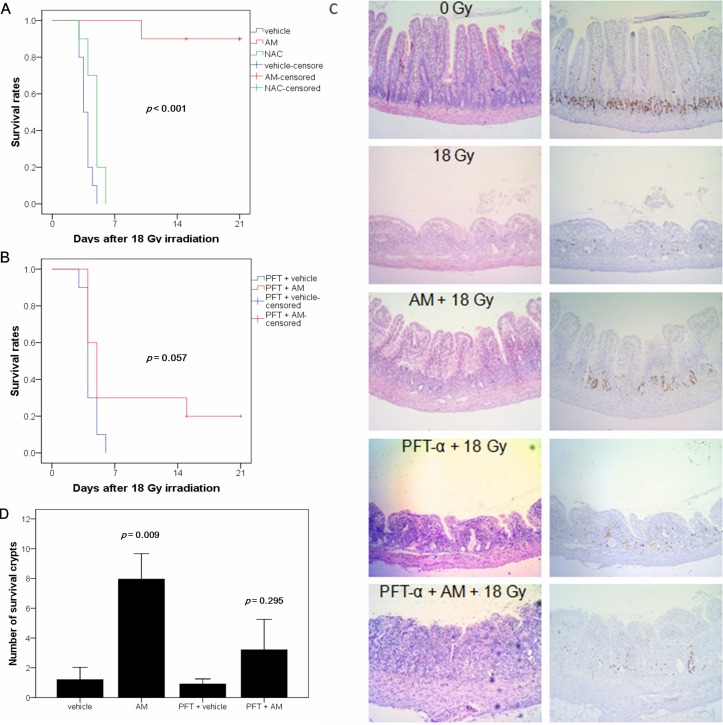
AM prevents radiation-induced lethal damage of the small bowel The radioprotective effect of AM on the small bowel was inhibited by a p53 inhibitor (PFT-α). Whole-abdominal irradiation was delivered to SD rats in five different groups (vehicle, AM, PFT-α, PFT-α plus AM, and NAC) (n=10 for each group). Survival times were observed, and Kaplan-Meier survival curves (n=10 for each group) were drawn for rats without **(A)** or with PFT-α **(B)**. Correlation between **(C)** mucosal damage (H & E stain) and BrdU uptake (IHC stain) 72 hours after irradiation was found in rats receiving different treatments. Original magnification, 100×. **(D)** Clonogenic crypt survival rates were calculated using BrdU administered 2 hours before rats were sacrificed and compared between the different groups (n=6 for each group). Error bars represent the standard error of the mean.

**Figure 3:**
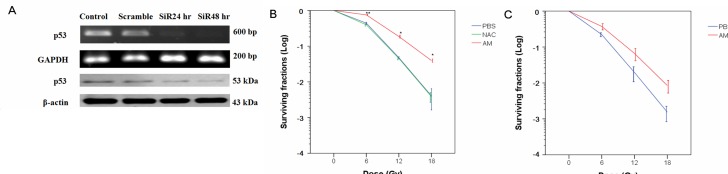
p53-dependent radioprotection of IEC-6 cells by AM **(A)** p53 siRNAs were delivered to IEC-6 cells, and mRNA and protein expression were determined at 24 or 48 hours after transfection. Whole cells were used for further experimentation for these conditions. **(B)** Clonogenic assays were performed in p53-proficient cells. Cells were pretreated with NAC (4 mM) or AM (4 mM) for 60 minutes, and the cells were then irradiated at 0, 6, 12, or 18 Gy for the clonogenic assays. Counting of colonies was performed 6 to 8 days after irradiation. **(C)** Clonogenic assays were assessed in p53 knockdown cells. IEC-6 cells were used for the clonogenic assays 48 hours after siRNA transfection. Cells were pretreated with AM (4 mM) or vehicle for 60 minutes and then irradiated. The clonogenic assays were performed for three independent experiments for each paired condition (with and without AM). Error bars represent the standard error of the mean. * *p* < 0.05; ** *p* < 0.01.

Because p53 mediates small bowel protection after irradiation [6,7,8] and AM has a radioprotective effect on the small bowel [3], we investigated whether the protective effect of AM is p53 dependent. We first assessed the survival rate of different groups of rats given lethal 18 Gy WAI. We also administered AM (200 mg/kg) and NAC (200 mg/kg), a compound with a similar effect to AM, to compare survival rates of rats treated with these compounds. No rats survived after 18 Gy WAI, and the median survival time was 3.5 days. NAC prolonged the median survival time of the rats to 5 days, although the overall survival rate remained at 0%. The overall survival rate in the AM group was 90%. Therefore, AM significantly rescued mortality in the rats compared with NAC (p < 0.001) (Figure 2A). To evaluate the role of p53 in AM-mediated protection of small-bowel damage after irradiation, we administered the p53 inhibitor PFT-α [13] 5 minutes before AM administration. The overall survival rate was 0% in the PFT-α group (median survival = 4 days) and 20% in the PFT-α/AM group (median survival = 5 days). The decrease in mortality induced by AM was not significant (*p* = 0.057) (Figure 2B). Next, we investigated whether the histopathology of the small bowels of the rats was correlated with the survival data from the different groups. Lethal irradiation induced severe mucosal damage (H & E stain) and no mucosal regeneration (BrdU uptake) 72 hours after 18 Gy WAI. AM alleviated the mucosal damage and enhanced recovery (Figure 2C) of the jejunum mucosa. Similar to the survival rates, the effect of irradiation on mucosal damage and recovery was less obvious in rats pretreated with PFT-α (Figure 2C). Quantitative assessments of the surviving crypts per circumflex were used to confirm the histopathologic findings. We found that AM increased the number of surviving crypts (*p* = 0.009) (Figure 2D). The effect of AM on surviving crypts was less obvious in rats that had been pretreated with PFT-α (*p* = 0.295) (Figure 2D). We next used rat crypt cells (IEC-6) to confirm the *in vivo* studies and further investigate these mechanisms *in vitro*. First, siRNA was used to knock down p53 in IEC-6 cells (Figure 3A), in which AM but not NAC increased clonogenic survival (Figure 3B). However, AM did not significantly improve clonogenic survival in IEC-6 cells that had been transfected with p53 siRNA (Figure 3C). These *in vivo* and *in vitro* results reveal that AM requires p53 to cause the observed radioprotective effects.

### AM increases p53 expression before but not after irradiation by delaying p53 degradation

**Figure 4:**
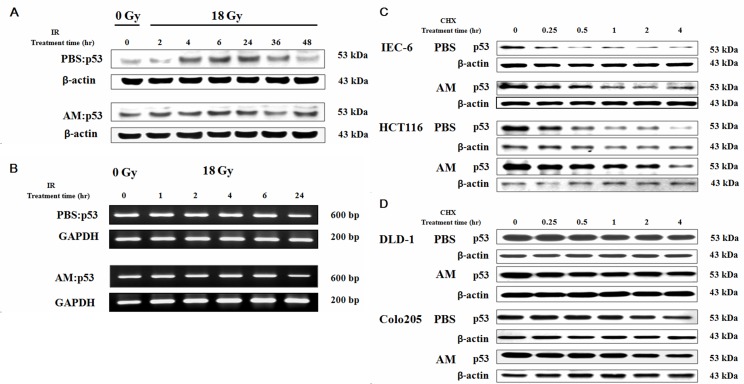
AM increases p53 expression when added before but not after irradiation by delaying p53 degradation but not inhibiting p53 transcription IEC-6 cells were treated with AM (4 mM) or vehicle (PBS) for 60 minutes, after which these compounds were washed out. Cells were irradiated using 18 Gy. Protein and mRNA levels of p53 were analyzed using **(A)** Western blotting and **(B)** RT-PCR, respectively, at different time points. The assay for p53 degradation in **(C)** p53 wild type cells (IEC-6 cells and HCT 116 cells) and **(D)** p53 mutant cells (DLD-1 cells and COLO 205 cells) was performed 60 minutes after AM (4 mM) or vehicle (PBS) administration, after which the AM and vehicle were washed out. After treatment with 50 μg/mL cycloheximide (CHX) for different durations, the cells were harvested, and Western blotting for p53 expression was performed.

Because the radioprotective effect of AM was p53 dependent, we further investigated whether AM could increase p53 expression before or after irradiation. IEC-6 cells were treated with AM or PBS for 60 minutes, and the cells were then irradiated and incubated for different lengths of time. We noted that AM increased p53 expression in IEC-6 cells before irradiation (Figure 4A). However, no further increase in p53 expression after irradiation was observed (Figure 4A). Increased p53 expression was noted 4 hours after irradiation and returned to baseline levels by 48 hours in cells that had not been treated with AM (Figure 4A). Therefore, we examined p53 transcription in untreated and AM-treated cells. Neither AM nor irradiation increased p53 mRNA expression (Figure 4B). Because enhancement of p53 protein expression by AM did not occur due to an increase in p53 mRNA expression, we considered that AM might affect p53 degradation. We used cycloheximide to inhibit protein synthesis in different types of intestinal cells to study p53 degradation. AM delayed p53 degradation in intestinal cells expressing wild type p53, including IEC-6 cells and HCT 116 cells (Figure 4C). The baseline levels of p53 were also higher in AM-treated IEC-6 and HCT 116 cells. However, AM did not affect the baseline levels of p53 and did not delay p53 degradation in intestinal cells expressing mutant p53, including DLD-1 and COLO 205 cells (Figure 4D).

### AM inhibits interactions between p53 and MDM2 in the nucleus

**Figure 5:**
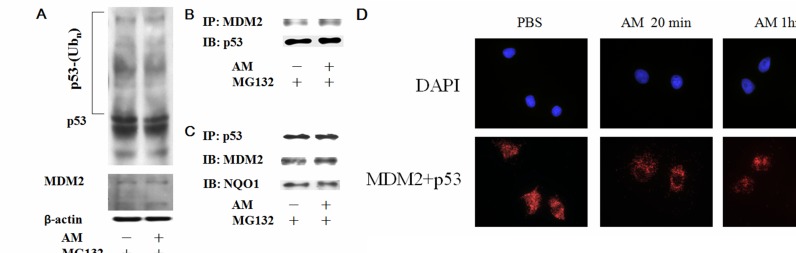
AM inhibits the interaction between MDM2 and p53 in the nucleus **(A)** A ubiquitination assay was performed to compare the effects of AM and PBS on cells. IEC-6 cells were transfected with HA-tagged ubiquitin and then treated with MG132 (20 μg/mL) for 4 hours to inhibit proteasome function. AM or PBS was then administered for 60 minutes. Western blots of p53 were performed to detect polyubiquitination of p53. **(B)** Immunoprecipitation (IP) for MDM2 and subsequent immunoblotting (IB) for p53 was performed using total cell lysates. IEC-6 cells treated with AM or PBS for 60 minutes were used for IP and IB. **(C)** IP for p53, followed by IB for MDM2 or NQO1, was performed using total cell lysates. **(D)** *In situ* PLA for p53 and MDM2 20 and 60 minutes after administration of AM or PBS.

Well-known molecules involved in p53 degradation include MDM2 [14,15], ubiquitin [15,16], and NAD(P)H quinone oxidoreductase 1 (NQO1) [17,18]. To investigate whether these markers are involved in the delay of p53 degradation by AM, we used immunoprecipitation (IP) and *in situ* proximity ligation assays (PLA) to examine the interaction between and these markers. After incubation with the proteasome inhibitor (MG132), AM did not change the degree of p53 polyubiquitination (Figure 5A). Using IP for MDM2, no difference in p53 immunoblotting was noted between the vehicle and AM groups (Figure 5B). In addition, no difference in MDM2 or NQO1 immunoblotting was noted between the vehicle and AM groups (Figure 5C). However, *in situ* PLA analysis demonstrated that AM decreased the interaction between p53 and MDM2 in the nucleus 20 minutes after AM administration (Figure 5D).

### AM enhances p53 accumulation in the nucleus but not the cytosol and increases transcriptional activity

**Figure 6:**
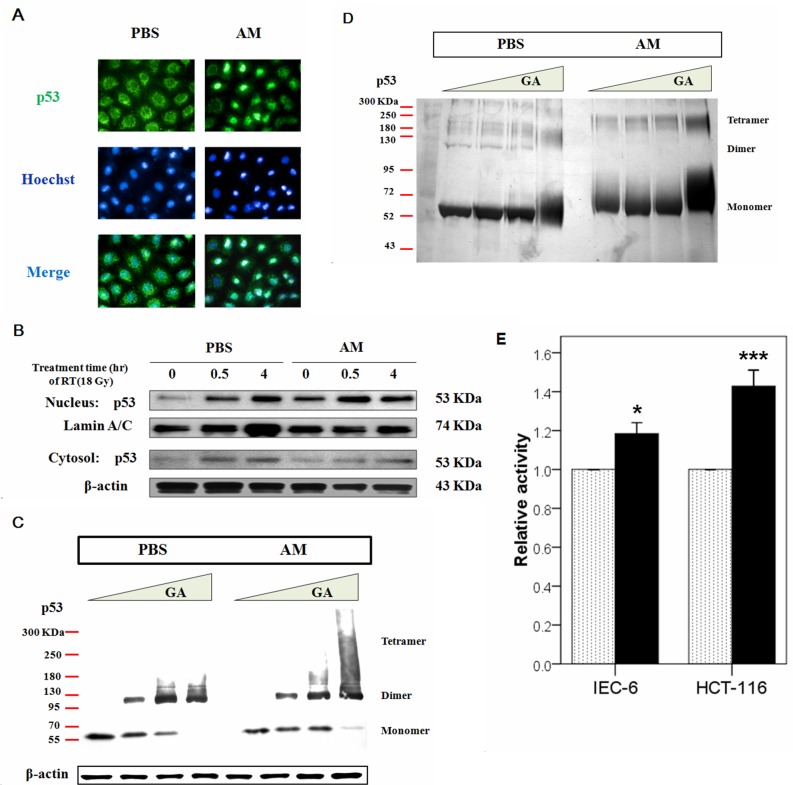
AM increases the nuclear but not cytosolic fraction of p53 **(A)** Immunofluorescent staining for p53 was performed in IEC-6 cells that had been treated with AM (4 mM) or vehicle (PBS) for 60 minutes, after which these compounds were washed out. Green color indicates p53 expression, and blue color indicates Hoechst nuclear staining. **(B)** Analysis of the nuclear and cytosolic fractions of p53 was performed between AM- and PBS-treated cells before and after irradiation. The internal control was lamin A/C for the Western blot assays. **(C)** Oligomerization assays were performed using glutaraldehyde (GA) for crosslinking. The relative amounts of p53 monomers, dimers, and tetramers, which exhibit different molecular weights, were determined using Western blotting. **(D)** An oligomerization assay in a cell-free system was performed. Purified human p53 (500 ng) (lanes 1-8) was pretreated with AM (lanes 5-8) for 60 minutes at 37°C and then incubated with 0% (lanes 1 and 5), 0.008% (lanes 2 and 6), 0.016% (lanes 3 and 7), or 0.09% (lanes 4 and 8) GA for 15 minutes at 37°C. Samples were analyzed by SDS/4-20% PAGE, followed by silver staining. **(E)** The transcriptional activity of p53 was compared between AM (black bar)- and vehicle-treated cells in IEC-6 and HCT 116 cells. Error bars represent the standard error of the mean. * *p* < 0.05, *** *p* <0.001.

Because the majority of p53 degradation occurs in the cytosol and our data revealed that AM did not affect the interactions between p53 and ubiquitin or p53 and NQO1, the export signals of p53 were investigated. To assess whether AM caused p53 accumulation in the nucleus, we used immunofluorescent microscopy to observe the location of p53 in AM-treated cells and found that AM enhanced the nuclear localization of p53 (Figure 6A). We also analyzed p53 expression in the nuclear and cytosolic fractions of cell lysates before and after irradiation. AM increased p53 expression in the nucleus but not the cytosol before irradiation (Figure 6B). However, AM did not significantly increase p53 expression in the nucleus 0.5 and 4 hours after irradiation compared with pre-irradiation levels. p53 tetramer formation plays a role in transcriptional activity [10]. Therefore, we investigated whether AM enhances p53 tetramerization and transcription. We used glutaraldehyde (GA), a protein crosslinker, to study p53 tetramerization [19] and found that AM increased p53 tetramer formation (Figure 6C). To investigate whether AM has a direct effect on p53 tetramerization, we used exogenous recombinant human p53 in a cell-free system and found that AM increased p53 tetramerization (Figure 6D). AM also enhanced the transcriptional activity of p53 in both IEC-6 and HCT 116 cells (Figure 6E).

### 14-3-3σ is involved in AM-mediated p53 nuclear accumulation and radioprotection

**Figure 7:**
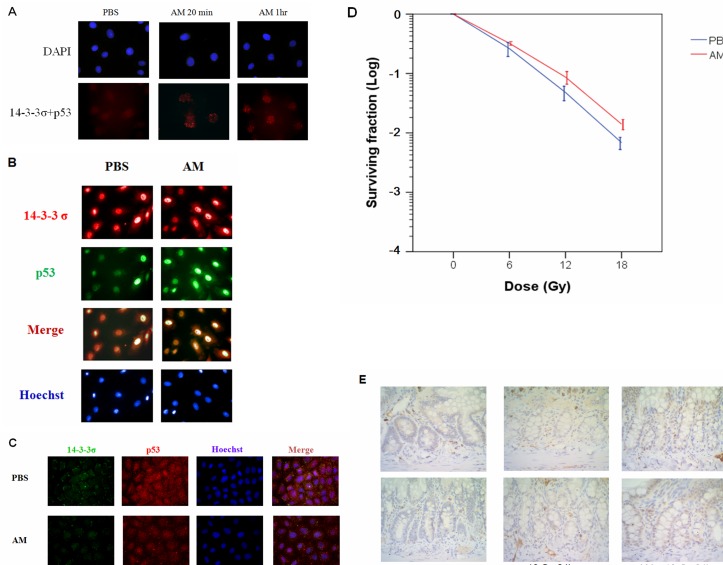
AM enhances the interaction between p53 and 14-3-3σ IEC-6 cells were treated with AM (4 mM) or vehicle (PBS) for 60 minutes before being washed out. **(A)** *In situ* PLA for p53 and 14-3-3σ was assessed for nuclear interactions. **(B)** Immunofluorescent staining for p53 (green) and 14-3-3σ (red) was assessed in IEC-6 cells with or without AM treatment. **(C)** Immunofluorescent staining for p53 (red) and 14-3-3σ (green) was assessed in 14-3-3σ-silenced IEC-6 cells with or without AM treatment. **(D)** Clonogenic assays were performed for radiosensitivity in ICE-6 cells in which 14-3-3σ had been knocked down. IEC-6 cells with stable clones containing 14-3-3σ shRNA were treated with AM or vehicle (PBS) 60 minutes before irradiation. The clonogenic assays were performed for three independent experiments for each paired condition (with and without AM). **(E)** IHC for 14-3-3σ expression in the crypts of the jejunum 24 hours after irradiation. Original magnification, 400×.

Our data suggested that AM-enhanced p53 nuclear accumulation and tetramerization might be related to inhibition of p53 and MDM2 interaction. One important molecule involved in these pathways is 14-3-3σ [11,12]. *In situ* PLA analysis was used to demonstrate that AM enhanced the interaction between p53 and 14-3-3σ (Figure 7A). We were interested in determining whether p53 could increase 14-3-3σ expression; however, our findings indicated that p53 did not increase the expression of this molecule (Figure 7B). To investigate the involvement of 14-3-3σ in p53-mediated radioprotection by AM, we used siRNA to silence 14-3-3σ and found that AM did not enhance p53 nuclear accumulation (Figure 7C). In IEC-6 cells treated with 14-3-3σ shRNA, AM did not significantly protect against radiation-induced cell death (Figure 7D). Furthermore, AM enhanced nuclear expression of 14-3-3σ in the crypts of the jejunum 24 hours after 18 Gy WAI (Figure 7E).

## DISCUSSION

Acute lethal radiation injuries are complex, and studies have demonstrated that total body irradiation typically presents with gastrointestinal (GI) and/or hematopoietic (HP) syndromes. The causes of death are dependent on the dose and types of tissues affected. In general, the LD_50_ of bone marrow is approximately 6-7 Gy. GI syndrome arises after an irradiation dose of 8 Gy or greater, and the threshold of the lethal dose is 15 Gy [20]. When the survival of an animal is used as an endpoint in a study, it is difficult to distinguish the cause of death as GI or HP syndrome using total body irradiation greater than 15 Gy. Therefore, WAI rather than total body irradiation is suitable for the study of radiation-induced lethal small bowel damage. The LD_50_ for rats is lower than that for mice [21]. The survival rate was previously reported as approximately 60% for mice receiving 16.95 Gy of abdominal irradiation [6]. In our experience, 12 Gy WAI resulted in reversible small bowel damage [22], and in the current study, 18 Gy WAI resulted in lethal and irreversible small bowel damage.

Drugs that can rescue lethal radiation injury are rare. The effectiveness of such drugs exists only at radiation doses less than 15 Gy [7,23,24] that do not induce lethal small bowel damage. The destructive mechanism of radiation may be via the attenuation of bone marrow more than via GI damage. Kirsch et al. found that p53 controls radiation-induced GI syndrome in mice independently of apoptosis [6]. In the study, tissue-specific inhibition of apoptotic signals could not protect against small bowel damage after irradiation [6]. An investigation into more effective drugs for the protection of lethal radiation-induced small bowel damage is attractive.

AM (WR-2721) is a phosphorylated prodrug that is dephosphorylated in tissues to its active form, WR-1025, to scavenge free radicals formed by radiation through membrane-bound alkaline phosphatase (AP) [4]. Earlier studies on the pharmacokinetic distribution of WR-2721 suggested that WR-2721 distributes in normal tissues rather than in tumors, resulting in selective normal tissue protection [2]. Although protection of the small bowel by AM has previously been investigated [3,5], the endpoints in such studies have typically relied on crypt survival and mucosal damage. Only one study presented a 54% survival rate for mice using 150 mg/kg of AM following 13 Gy of total body irradiation [23]. However, the dose of AM in that study was not very effective [3]. In the present study, we used 18 Gy as the lethal dose for abdominal irradiation. Although this dose was higher than that in most studies investigating radiation-induced small bowel damage, AM could rescue most of the irradiated rats from death.

AM can scavenge hydroxyl radicals but not superoxide in vitro [25] and can scavenge ROS in vivo [26]. To exclude the effect of general free radical scavenging in this study, we chose NAC as a control, given its reported role as an antioxidant and a strong scavenger of hydroxyl radicals [27]. Because 1 Gy produces 0.27 μM hydroxyl radicals in neutral water [28], it is possible that 18 Gy of radiation induces an amount of hydroxyl radicals (estimated as 4.86 μM) that is too small to detect large differences compared with 0 Gy treatment in the present experiments. NAC and AM similarly reduced the levels of hydroxyl radicals after 18 Gy irradiation. Although the initial DNA damage was similar between AM and NAC, the survival rates of IEC-6 cells and rats were better following AM treatment than for NAC treatment. These results are consistent with a study that reported that WR-1065 (4 mM) but not NAC (4 mM) protected human microvascular endothelial cells after 8 Gy irradiation [29]. In addition, the data suggested that PFT-α and p53 knockdown attenuated the effect of AM. Under conditions of p53 inhibition, the radioprotective effect of AM was considered to be due to ROS scavenging. The effect of p53 induction on survival might be more involved than ROS scavenging alone. Therefore, ROS scavenging-independent effects of AM may play an important role in radioprotection based on data examining p53 inhibition. Our results support the hypothesis that p53 mediates radiation-induced small bowel damage [6].

Surprisingly, p53 accumulated in IEC-6 cells 60 minutes after AM administration. This finding is consistent with a study in MCF-7 cells that revealed that AM enhanced binding of p53 to DNA [30]. AM also increased p53 expression [30,31], with this enhancement occurring as early as 30 minutes and persisting to 90 minutes. Cassatt et al. found that WR-1065 levels in the small intestine of rats reached a plateau at approximately 15 minutes following the intravenous administration of AM and remained at that level for approximately 90 minutes [32]. Because the uptake of AM into cells occurs within 15 minutes and intracellular levels of AM are maintained for up to 90 minutes [33], p53 expression had sufficient time to increase before irradiation began. Our data confirmed the results of these studies. In our study, the incubation time of AM was 1 hour for IEC-6 cells. Even at 2 hours after 18 Gy irradiation, p53 was not significantly induced. However, pretreatment with AM increased p53 expression in cells 2 hours after irradiation compared with no AM pretreatment. Increased p53 expression by AM may explain the radioprotective effect of AM because p53 mediates radiation-induced small bowel damage [6]. Hence, AM may mimic p53 overexpression in vitro and in vivo based on findings from the present study. An in vivo study [13] using PFT-α to inhibit p53 transactivation confirmed that AM acts via the activation of p53 downstream genes. Induction of p53, by causing arrest [31], protects normal cells from cycle-dependent chemotherapy or radiation. At the same time, these p53-inducing agents did not protect cancer cells with mutant p53 [34-37]. Such selective protection can extend therapeutic window.

Our investigation of the potential rapid induction of p53 by AM examined known pathways of degradation of p53. However, NQO1 and ubiquitin were not involved in the delay of p53 degradation by AM. We suggest that the dynamics of p53 synthesis and degradation in the cytosol is not changed by AM. Therefore, the inhibition of p53 export may be the mechanism underlying the effect of AM on p53. The nuclear but not cytosolic accumulation of p53 supports our hypothesis. Because tetramerization of p53 leads to p53 transcriptional activation [10], it was important to investigate the role of AM in p53 tetramerization. Our data revealed that p53 tetramerization and transcriptional activity were increased by AM. MDM2 is associated with p53 export [19,38], and the interaction of MDM2 with p53 was inhibited by AM in our results. 14-3-3σ can inhibit the interaction between MDM2 and p53 and increase p53 stabilization [11,12]. 14-3-3σ is required to prevent mitotic catastrophe after DNA damage [39]. We noted that AM enhanced the interaction between 14-3-3σ and p53. Therefore, 14-3-3σ may play a role in AM-medicated regulation of nuclear p53 accumulation. To investigate the role of 14-3-3σ in AM-mediated radioprotection, we knocked down 14-3-3σ and found that the knockdown compromised the effects of AM, including nuclear accumulation of p53, and increased clonogenic survival. Even in a cell-free system, AM enhanced p53 tetramer formation. Taken together, our data suggest that AM first increases tetramer formation and subsequently enhances the interaction between p53 and 14-3-3σ, which normally inhibits p53 degradation and enhances p53 transcriptional activity. Subsequent radioprotection by increased p53 expression prior to lethal small bowel irradiation is an important mechanism induced by AM. It is worth identifying specific drugs that enhance the p53/14-3-3σ interaction in the nucleus and thus alleviate small bowel damage.

## METHODS

### Cell culture and drug administration

IEC-6 (CRL-1592), HCT 116 (CCL-247), COLO 205 (CCL-222), DLD-1 (CCL-221), and HEK293T (CRL-11268) cells were purchased from the American Type Culture Collection (ATCC). Cells were grown in suggested media (Gibco Life Technologies, USA) with 10% fetal bovine serum (FBS) (Gibco Life Technologies, USA) and antibiotics (Gibco Life Technologies, USA) at 37°C under an atmosphere of 5% CO_2_. The media was changed every 3 days, and cells were passaged by 0.25% trypsin-EDTA (Gibco Life Technologies, USA). Subconfluent cancer cells were cultured in a 25T flask for further experiments. The combination of AM (4 mM) and AP (1 U/ml) to form active WR1065 was based on a study by Smoluk et al. [40]. Washout of AM and AP was performed 60 minutes after drug administration or irradiation.

### Determination of 8-hydroxy-2′-deoxyguanosine (8-OHdG) levels by flow cytometry

The manufacturer's protocols were followed for the following experiments. Briefly, 1×10^5^–5×10^5^ cells were harvested, transferred to polystyrene flow cytometry tubes, washed with PBS, pelleted, and resuspended in 1 mL of PBS. After 0.5 mL of 2% paraformaldehyde was added, the solution was placed on ice for 15 minutes. The cells were washed twice with 0.1% BSA solution, resuspended in 1 mL of ice-cold 90% ethanol to permeabilize the cells, and stored at −20°C until analysis. After washing, nonspecific sites were blocked by incubation with blocking solution at 37°C for 1 hour. After washing, diluted concentrations (1:50) of primary antibody specific for 8-hydroxy-2′-deoxy-guanosine [N45.1] (ab48508, Abcam Cambridge Science Park, Cambridge, UK) were incubated with the cells for 1 hour in the dark at 37°C. After additional washing, diluted concentrations (1:100) of FITC-conjugated antibodies specific for KLH (ab34766, Abcam Cambridge Science Park, Cambridge, UK) or negative control anti-rabbit FITC-conjugated IgG antibodies were incubated with the cells for 30 minutes in the dark at 37°C. After washing, the cells were rinsed, resuspended, and analyzed with a fluorescence-activated cell sorter (FACSCalibur system, Becton Dickinson). Fluorescence readings were obtained for 10^4^ cells for each set of experiments and analyzed with CellQuest Software.

### siRNA transfection

Cells were seeded in media without antibiotics. After 24 hours, 10 μL Lipofectamine 2000 (Invitrogen) was incubated for 5 minutes with 240 μL Opti-MEM (Invitrogen). Next, 0.2 nmol (100 nM) of non-specific (Invitrogen), p53-specific siRNA (sc-45917, Santa Cruz biotechnology, CA), or 14-3-3σ-specific siRNA (SR503571, OriGene Technologies, Rockville, MD) was resuspended in 250 μL Opti-MEM and combined with Lipofectamine 2000. After 20 minutes, 500 μL of the siRNA-Lipofectamine 2000 mix was added to cells with 1.5 mL Opti-MEM. Six hours later, complete growth media was added.

### Reverse transcription-polymerase chain reaction (RT-PCR)

IEC-6 cells were resuspended using the REzolTM C&T reagent (Protech Technologies, Taiwan), and total cellular RNA was extracted according to the manufacturer's protocol. The RNA samples were assessed for quantification and purity by A260/A280 absorption, and RNA samples with ratios greater than 1.8 were stored at −80°C for further analysis. RT-PCR was performed using a MMLV-Reverse Transcriptase kit (EPICENTRE® Biotechnologies, Wisconsin, USA). To synthesize complementary DNA (cDNA), 2 μg RNA was resuspended in 14.5 μL of diethylpyrocarbonate-treated water, 1.5 μL of oligo(dT) primer, 3 μL of 0.1 M DTT, 3 μL of 10× RT buffer (0.5 M Tris-HCl, 0.1 M MgCl_2_, and 0.75 M KCl, pH 8.3), 6 μL of 2.5 mM dNTP, 0.5 μL of RNase inhibitor, and 1 μL of Moloney murine leukemia virus reverse transcriptase (MMLV RT). The reaction mixture was incubated for 60 minutes at 37°C to synthesize the DNA strand, and the reaction was stopped by denaturing the enzyme at 85°C for 5 minutes. One microgram of cDNA was used for PCR amplification (Promega, Madison, WI) in a reaction volume of 20 μL containing 2 μL 10x PCR Buffer, 0.25 μL 10 mM dNTP, 0.2 μL Taq, 0.5 μL of each specific primer, and sterile water. The conditions for the rat p53 RT-PCR experiments were as follows: the reaction mixture was heated to 94°C for 2 minutes, and the amplification was performed for 25 cycles of 94°C for 1.5 minutes, 60°C for 50 s, and 72°C for 50 s on a thermal cycler; and the reaction mixture was then heated at 72°C for 5 minutes. The rat GAPDH conditions were as follows: the reaction mixture was heated to 94°C for 2 minutes, and the amplification was performed for 25 cycles of 94°C for 1.5 minutes, 54°C for 45 s, and 72°C for 50 s on a thermal cycler; and the reaction mixture was then heated at 72°C for 5 minutes. The primers used for the rat p53 experiments were as follows: forward: 5′-TCT GTC ATC TTC CGT CCC TTC TC-3′, and reverse: 5′-AAC ACG AAC CTC AAA GCT GTC CCG-3′. Primers for rat glyceraldehyde-3-phosphate dehydrogenase (GAPDH): forward: 5′-CCA TGG AGA AGG CTG GGG-3′, and reverse: 5′-CAA AGT TGT CAT GGA TGA CC-3′. Amplified DNA (10 μL of the PCR mixture) was resolved on a 1.5% agarose gel by electrophoresis and visualized under UV illumination.

### Whole cell protein extract

Cells were lysed in RIPA buffer (0.1% SDS, 1% NP-40, 0.5% deoxycholate, 150 mM NaCl, 1 mM EDTA, 0.5 mM EGTA, and 50 mM Tris-Cl, pH 8) with 1 mM PMSF, 8.5 μg/mL aprotinin, 2 μg/mL leupeptin, 1× complete mini protease inhibitor cocktail (Roche), and 1× phosphatase inhibitor cocktail (Pierce) following the standard protocol.

### Immunoprecipitation and Western blotting

Co-IP was performed using the Catch and Release v2.0 Reversible Immunoprecipitation System (Millipore, Billerica, MA) according to the manufacturer's instructions. Binding of p53 to MDM2 was detected by immunoprecipitation (IP) analysis. In brief, whole cell extracts for immunoprecipitation experiments were carried out using a primary antibody to MDM2 (SMP 14; sc-45917, Santa Cruz Biotechnology, CA) or p53 (FL-393; sc-6243, Santa Cruz biotechnology, CA). Typically, 500 μg of lysate was incubated with 4 μg of MDM2 or p53-specific antibody overnight at 4°C. The immunoprecipitates or protein extracts (50 μg) from cells were heated at 94°C for 3 minutes, resolved by 10% SDS-PAGE, and electrotransferred to PVDF membranes using semi-dry transfer. The membranes were blocked for 1 hour at room temperature with PBS containing 5% milk powder, incubated with primary antibody diluted for p53 (1C12; #2524, cell signaling technology), MDM2 (SMP 14; sc-45917, Santa Cruz Biotechnology, CA), ubiquitin (ab7780, Abcam, Cambridge, MA), or 14-3-3σ (E-11; sc-166473, Santa Cruz Biotechnology, CA) and subsequently incubated with anti- β-actin horseradish peroxidase-conjugated secondary antibody (diluted concentration 1:5000) at room temperature for 50 minutes. Immunoblots were then probed with the mouse monoclonal anti-p53 antibody. MDM2 expression was determined using the same membrane after stripping the immune complexes used for the detection of p53.

### Irradiation and clonogenic assay

Cells under different conditions (scrambled RNA, siRNA, or overexpression) were irradiated in 25T flasks. For the clonogenic assay, 0, 12, and 18 Gy were delivered using a linear accelerator. Cells (100-10,000/well, according to the radiation dose) were plated in six-well plates immediately following irradiation. At the 1 to 2 weeks following the irradiation, glutaraldehyde (6.0% v/v) and crystal violet (0.5% w/v) were added to fix and stain the colonies, respectively. Cells were counted using a stereomicroscope. A colony was considered to be surviving when 50 or more cells were counted. Normalization to 0 Gy in each condition was performed according to the plating efficiency (PE). The surviving fraction equaled the number of colonies/(number of cells plated × PE). The survival curve was drawn according to the survival rate (log) and dose.

### Animals, drug administration, and irradiation

Male Sprague-Dawley (SD) rats (300-400 g) were used for lethal irradiation. The animal protocol was approved by the animal care committee at our institution. Rats were subjected to 18 Gy whole-abdominal irradiation (WAI). Vehicle, AM (200 mg/kg, i.p.), or NAC (200 mg/kg, i.p.) was administered 30 minutes before WAI. PFT-α(2.2 mg/kg, i.p.) was administered 5 minutes before AM administration. The details of the WAI were described in a previous study [22]. To determine 8-OHdG and 14-3-3σ expression, rats under different conditions were killed 24 hours after WAI, and a segment of the distal jejunum was obtained. For the determination of crypt survival, BrdU (100 mg/kg, i.p) was administered 70 hours after WAI; additionally, rats under different conditions were killed 72 hours after WAI, and a segment of the distal jejunum was obtained. For the survival observations, the animals were fed normally and observed.

### Immunohistochemistry (IHC)

The paraffin sections (3 μm-thick) were deparaffinized, rehydrated, and subjected to antigen retrieval. Antigen retrieval was achieved by incubation in a water bath at 95°C for 30 minutes (for 8-OHdG or 14-3-3σ) or by incubation with 1 N HCl for 60 minutes at 40°C, followed by treatment with 0.1% trypsin for 20 minutes (for BrdU). To block endogenous peroxidase activity and nonspecific binding sites, the sections were incubated with 3% hydrogen peroxide for 10 minutes. The sections were incubated with primary antibodies directed against 8-OHdG (ab48508, Abcam, Cambridge, MA), 14-3-3σ (sc-166473, Santa Cruz), and BrdU (MAB5404, Chemicon) at dilutions of 1:3000, 1:2000, and 1:1000, respectively, for 60 minutes. After washing, the sections were incubated with a horseradish peroxidase (HRP)-conjugated anti-rabbit/mouse detection system (Dako REAL EnVision, Dako Inc., Denmark) for 30 minutes and visualized with 3,3′-diaminobenzidine (DAB). As a negative control, another set of sections stained without primary antibodies were subjected to incubation with secondary antibodies, DAB treatment, and hematoxylin staining.

### Preparation of nuclear and cytoplasmic fractions

Nuclear and cytoplasmic fractions were prepared using Cayman's Nuclear Extraction Kit (Cayman Chemical Company, Ann Arbor, MI; item no. 10009277). The purity of the fractions was determined using β-actin as a cytosolic marker and lamin A/C as a nuclear marker.

### Protein decay analysis

Cells were pre-treated with vehicle or 4 mM AM in addition to AP (1 U/mL) for 60 minutes, and the medium was then replaced with fresh medium. After treatment with 50 μg/mL cycloheximide (CHX) (Sigma–Aldrich, St. Louis, MO) for different durations (15 to 240 minutes), the cells were harvested, and the whole cell lysates were processed for immunoblot analysis of p53 and β-actin.

### Oligomerization assay

IEC-6 cells and HCT 116 cells were treated with or without 4 mM AM in addition to AP (1 U/ml) for 60 minutes. Equal amounts of cell lysates were treated without or with glutaraldehyde at a final concentration of 0.008%, 0.016%, or 0.09% for 2 to 5 minutes at 37°C. The reactions were terminated by the addition of 10 μL of 1 M Tris-HCl, pH 8.0. Cross-linked proteins were solubilized by the addition of an equal volume of 4x XT sample buffer, and SDS-PAGE was conducted in a 6% to 12% gel. Samples were then analyzed by Western blot analysis for p53 dimerization and tetramerization.

### Ubiquitination assay

IEC-6 cells were transfected with plasmids encoding HA-tagged ubiquitin and pcDNA3 encoding WT MDM2. The cells were treated with 20 μg/mL MG132 (Sigma) for 4 hours, harvested, washed twice with PBS (pH 7.4), and lysed in 150 μL of RIPA-buffer. p53-ubiquitinated proteins were targeted with an anti-p53 antibody and subjected to 10% SDS-PAGE, followed by Western blot analysis.

### Immunostaining

Cells samples were immunostained to visualize p53, 8-OHdG, MDM2, and 14-3-3σ according to the manufacturer's instructions, as previously described (double immunofluorescence-simultaneous protocol, Abcam, Cambridge, MA). Next, 5 × 10^3^ cells were placed onto 3 cm coated coverslips in dishes for cell culture. The cells were then fixed in 4% paraformaldehyde for 15 minutes and washed two times with ice-cold PBS buffer for 5 minutes each. The samples were incubated for 10 minutes in PBS containing 0.5% Triton X-100 for permeabilization, and the cells were then washed in PBS three times for 5 min. The cells were incubated with 2% BSA in PBST for 30 minutes to block unspecific binding of the antibodies. The cells were incubated in either a primary antibody such as p53 (PAb 240) (ab26, Abcam, Cambridge, MA), p53 (1C12) (Alexa Fluor-488 conjugate,#2015, Cell signaling technology, Mölndal, Sweden), 14-3-3σ (N-14) (sc-166473, Santa Cruz Biotechnology, CA) or a mixture of two primary antibodies in 1% BSA in PBST in a humidified chamber for 1 hour at room temperature or overnight at 4°C. The solution was decanted, and the cells were washed three times in PBST for 5 minutes each. The cells were incubated with either a secondary antibody or a mixture of two secondary antibodies produced in animals of different species with two different fluorochromes (i.e., anti-mouse Texas Red-conjugated and anti-rabbit FITC-conjugated antibodies) in 1% BSA for 1 hour at room temperature in the dark. The mixture containing the secondary antibodies was decanted, and the cells were wash three times with PBST for 5 minutes each in the dark. The cells were incubated with 1 μg/mL Hoechst 33342 for 3 minutes and then rinsed with PBS. Finally, the cells were mounted on a coverslip with a drop of mounting medium. The cells were observed using a fluorescence microscope (Axio Observer Z1, Carl Zeiss MicroImaging, Inc., Welwyn Garden City, UK) and photographed using an integrated camera with the appropriate filter for detection.

### Stable knockdown of 14-3-3σ

We used HEK293T cells for the transient transfections and for the production of all recombinant retroviruses following the manufacturer's instructions (HuSHTM shRNA plasmid panels application guide). Briefly, the packing cells were allowed to reach 70-80% confluency and were cotransfected with 1 μg of *Rattus norvegicus* 14-3-3σ (LOC298795) shRNA plasmid (constructs were in the retroviral pGFP-V-RS vector (SRTG702077, OriGene Technologies, Rockville, MD) with TurboFect in vitro Transfection Reagent (Fermentas). The cells were incubated with fresh media the next day, and on day two post-transfection we collected the media from the cultures and either centrifuged the media at 2000 x g for 5 minutes or passed it through a 0.45 μm filter to remove cell debris. The supernatant was frozen at −80°C or directly used as a viral stock to determine viral titers. The viral titers were calculated 72 h after transfection by counting the number of GFP-expressing foci divided by the dilution factor. For viral infections, IEC-6 cells were plated and had reached approximately 50% confluency after 24 hours. The entire amount of viral stock and polybrene (8 μg/mL) in growth medium was added directly onto target cells and incubated at 37°C in 5% CO_2_. At 24 hours post-infection, the medium was replaced with fresh medium supplemented with 10% FBS. Puromycin (0.5-1 μg/mL) was used for selection; at the lowest drug concentration, puromycin resulted in massive cell death after 3 days and killed all the cells within 2 weeks. IEC-6 cells were plated into 96-well plates (1 × 10^3^ cells per well) in limiting dilutions and allowed to adhere for 16 hours. A selective medium with 400 to 600 ng/ml of puromycin was added to the stable cell lines the next day.

### Comet assay

Procedures were followed according to the manufacturer's instructions of the CometAssay kit (Trevigen Inc., Gaithersburg, MD, USA) as described previously [41]. Following electrophoresis, the slide was stained with SYBR Green (Molecular Probes, Eugene, OR, USA) diluted 1:10,000 in 10 mM Tris-HCl, pH 7.5, 1 mM EDTA. The images were observed and captured by fluorescence microscopy (Axio Observer Z1, Carl Zeiss MicroImaging, Inc., Welwyn Garden City, UK) with a 490 nm filter.

### *In situ* proximity ligation assay (PLA)

We used a Duolink^®^ reagent kit (Olink Biosciences) to investigate *in situ* protein-protein interactions. The procedures for the administration of primary antibodies (p53 versus MDM2 or p53 versus 14-3-3σ), PLA probes, hybridization, ligation, amplification, detection, and mounting were performed according to the manufacturer's protocol. The images were captured using a fluorescence microscope (Axio Observer Z1, Carl Zeiss MicroImaging, Inc., Welwyn Garden City, UK).

### p53 transcription factor assays

Commercial kits (TransAM p53 Transcription Factor Assay Kits, Active Motif, North America) were used to quantify p53 activation according to the manufacturer's protocol. Briefly, nuclear extracts were placed in a 96-well plate with immobilized oligonucleotides containing the p53 consensus binding site. A primary antibody against an epitope of p53 that is exposed upon DNA binding and a secondary antibody conjugated to HRP were added to provide a chemiluminescent readout, and the results were quantified using a spectrophotometer (Multiskan FC Microplate Photometer, Thermo Scientific) at 450 nm with reference wavelength of 655 nm.

### Statistics

Comparisons of samples in the clonogenic assays and comparisons of p53 transcriptional activity of each pair of samples were performed using paired t-tests. The number of surviving crypts in small bowels between the AM and no AM groups was compared using the Student's t-test. Survival analysis was calculated by the Kaplan-Meier method, and significant differences were tested by a log-rank test. A p value <0.05 was considered statistically significant. Statistical analyses were performed using the Statistical Package for Social Sciences, version 17.0 (SPSS, Chicago, IL, USA).
